# Reanalysis of Epidemiological Investigation of Cancer Risk among People Residing near Nuclear Power Plants in South Korea

**DOI:** 10.3390/ijerph15030481

**Published:** 2018-03-09

**Authors:** Jeong-Min Kim, Myoung-Hee Kim, Young-Su Ju, Seung-sik Hwang, Mina Ha, Bong-Kyu Kim, Kyung Ehi Zoh, Domyung Paek

**Affiliations:** 1 Department of Occupational and Environmental Medicine, Cheongju Medical Center, Cheongju 28547, Korea; juc28ten@yahoo.co.kr; 2 People’s Health Institute, Seoul 07004, Korea; hongsili@gmail.com; 3 Department of Occupational & Environmental Medicine, Hallym University Sacred Heart Hospital, Hallym University College of Medicine, Anyang 14068, Korea; zorro@hallym.ac.kr; 4 Department of Public Health Science, School of Public Health, Seoul National University, Seoul 08826, Korea; cyberdoc@snu.ac.kr; 5 Department of Preventive Medicine, Dankook University College of Medicine, Cheonan 31116, Korea; minaha00@gmail.com; 6 Department of Occupational and Environmental Medicine, School of Public Health, Seoul National University, Seoul 08826, Korea; bkbk2@hanmail.net (B.-K.K.); kezoh@snu.ac.kr (K.E.Z.); 7 Institute of Health and Environment, Seoul National University, Seoul 08826, Korea

**Keywords:** nuclear power plant, thyroid cancer, detection bias, South Korea

## Abstract

*Background*: A 20-year follow-up study on cancer incidence among people living near nuclear power plants in South Korea ended in 2011 with a finding of significantly, but inconsistently, elevated thyroid cancer risk for females. Reanalysis of the original study was carried out to examine the dose–response relationship further, and to investigate any evidence of detection bias. *Methods*: In addition to replicating the original Cox proportional hazards models, nested case-control analysis was carried out for all subjects and for four different birth cohorts to examine the effects of excluding participants with pre-existing cancer history at enrollment. The potential for detection bias was investigated using the records of medical utilization and voluntary health checks of comparison groups. *Results*: The overall risk profile of the total sample was similar to that of the original study. However, in the stratified analysis of four birth cohorts, the cancer risk among people living near nuclear power plants became higher in younger birth cohorts. This was especially true for thyroid cancers of females (hazard ratio (HR) 3.38) and males (HR 1.74), female breast cancers (HR 2.24), and radiation-related cancers (HR 1.59 for males, HR 1.77 for females), but not for radiation-insensitive cancers (HR 0.59 for males, HR 0.98 for females). Based on medical records and health check reports, we found no differences between comparison groups that could have led to detection bias. *Conclusions*: The overall results suggest elevated risk of radiation-related cancers among residents living near nuclear power plants, controlling for the selective survival effect. This is further supported by the lack of evidence of detection bias and by records of environmental exposure from radiation waste discharge.

## 1. Introduction

The first nuclear power plant (NPP) in South Korea opened in 1978 in Kori, Gyeongsang Province; three more NPPs opened in southern coastal areas through the 1980s. In 1989, owing to mounting questions regarding birth defects among children of employees, the Korean government decided to conduct an epidemiologic study on the impacts of the NPPs. Voluntary participants were recruited at all four NPP sites from 1992 to 2006, with two control groups from near and remote areas. Cancer development was followed up through 2008, and the final report from the original study was submitted in 2011. The major findings were published in 2012 [1].

One of the major findings was the significantly elevated risk of thyroid cancers among females residing near NPPs compared with those in control groups. However, the original authors considered that result irrelevant, commenting that there was no statistically significant difference in male thyroid cancers and that they could not find a dose–response pattern among females. They concluded that, “there is no epidemiological or causal evidence for increased risk of cancer due to radiation from NPPs”. However, the possibility that bias was induced by excluding cancer cases present at enrollment among people residing near the NPPs was raised in a series of correspondences [2,3,4]. The baseline surveys were performed 14 to 28 years after the NPPs had opened. The original authors’ contention that there was no need for further epidemiologic study was also challenged [5].

In this study, we reanalyzed the original data with permission from the Korea Foundation of Nuclear Safety, under the control of the Nuclear Safety and Security Commission of Korea. In this reanalysis, in addition to replicating the original analysis, we examined different models and utilized different analysis methods, including nested case-control analysis. The major focus of the reanalysis was to examine the dose–response relationships for various subgroups, and to find any evidence of detection bias, which the original authors claimed could have been caused by more frequent use of thyroid scans among people residing near NPPs.

## 2. Methods

**Figure A1:**
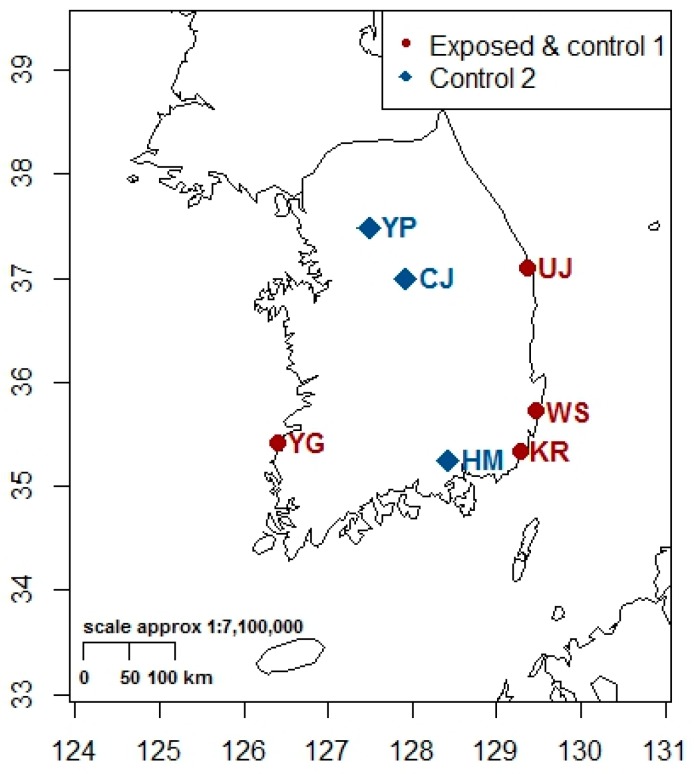
Locations of study areas; Kori (KR), Wolsong (WS), Uljin (UJ), and Yeonggwang (YG) for exposed and control 1 areas; Yangpyeong (YP), Chungju (CJ), and Haman (HM) for control 2 areas.

It is advised to read the original paper to figure out study populations [1]. A map of South Korea was included in the Appendix A (Figure A1) to indicate the locations of the study areas.

### 2.1. Data and Variables

We obtained the final version of data used for tabulations of the original report. However, it was a trimmed version of the original raw data. The records of 54,508 participants had been deleted because of insufficient information, and further 377 participants’ records had been excluded because of preexisting cancers identified at the baseline survey. The final version of the data included records of 11,367 participants living near NPPs and 24,809 participants residing in control areas, all aged 20 years and older.

The data included demographic characteristics and medical histories, but some covariates that we believed to be important, such as occupation or detailed residential address, were not provided. Clinical laboratory indicators measured at the baseline health examination survey were also unavailable.

Exposure status was defined in the same way as in the original study: subjects residing within 5 km of NPPs were categorized as ‘exposed’; subjects residing 5–30 km from NPPs were categorized as ‘unexposed’ or ‘control 1’; and subjects residing more than 30 km from NPPs were categorized as ‘unexposed’ or ‘control 2’.

The primary outcome was cancer development during the follow-up period. As per the guidelines of the International Agency for Research on Cancer (IARC), cancers of the following organs were categorized as ‘radiation-related’: brain (central nervous system), bone, skin (excluding melanoma), nose and sinus, lung and airway, thyroid, breast, salivary gland, esophagus, stomach, colon and rectum, liver and biliary tract, kidney, and bladder. Leukemia (excluding chronic lymphocytic leukemia) was also included in this category [6]. In the original study, follow-up was carried out only once at the end of the study, and no information about when and where was available for those lost to follow-up (LTFU). In our analysis, LTFUs without any evidence of follow-up were removed from the dataset, as we could not determine the time of LTFU which is required for survival analysis.

One participant with eosinophilia was categorized erroneously as a cancer patient in the original dataset, and, as we could not confirm the diagnosis, the record was also removed from the dataset.

### 2.2. Statistical Analysis

Since only about one-third of the total recruited subjects were included in the study, the effects of such deletion should have been examined directly; unfortunately, we were not given the relevant data required to do so. Instead, we attempted to examine the effect of selection indirectly, especially the removal of participants with pre-existing cancer history, on the study outcomes for the remaining participants of different age groups by dividing the entire cohort into four birth cohorts of equal sizes by birth year: 1901–1934, 1935–1942, 1943–1955, and 1956–1984.

Cohort analysis: Age-standardized rates (ASR) were calculated based on the age and sex structures of Segi’s world standard population [7] for three comparison groups. Out of the follow-up period of 1992 to 2008, nation-wide cancer incidence is available from 1999, and the standardized incidence ratio (SIR) based on indirect standardization with national reference was calculated only for the period of 1999 to 2008. Group-specific annual trends of ASRs were examined by the LOWESS (locally weighted scatterplot smoothing) method [8]. The incidence of thyroid cancer was particularly high at the Yeonggwang County NPP site. To achieve a comprehensive investigation of the effects, we plotted LOWESS lines both including and excluding records from Yeonggwang County.

The effect of exposure on cancer development was examined using hazard ratios estimated from Cox proportional hazards models, controlling for covariates including smoking, alcohol drinking, liver disease history, education, physical activities, family cancer history, body mass index, and medical radiation exposure. In creating the final models, we considered covariates which were missing for less than 10% of subjects. Family history of cancer was missing for about 35% of subjects and was not included in the final model. With few covariates available, we attempted to maintain inclusive models, first by mandating the inclusion of known risk factors (as determined by the IARC), then using backward elimination, and finally setting significance criteria at *p* < 0.2 for inclusion and *p* < 0.1 for remaining. We attempted to adjust for time-dependent covariates by including two out of the three time variables: exposure duration, age, and calendar period. Finally, stratified analyses were carried out for four different birth cohorts. In the end, six different Cox proportional hazards models for developing cancers were examined using the same adjustments over exposure periods and stratified into four birth cohorts.

We found that the effects were more-or-less robust among different models, and we chose to present the results of the models adopted in the original study with age as the main time varying variable and calendar year as covariate for comparative purposes rather than any particular advantage.

Nested case-control analysis: To test the robustness of the cohort analysis, we carried out a nested case-control analysis. The following six groups of incident cancer patients were selected as case groups: thyroid cancers, female breast cancers, male stomach cancers, male liver cancers, radiation-related cancers [6], and total cancers. For each case, four controls were selected randomly from the study subjects from exposed and two unexposed areas, matched by sex, age at diagnosis, and education (primary school, middle school, high school, college or more, missing). The number of controls was allocated proportionally according to the size of the study population for each area (31.4% for exposed areas, 28.5% for control 1 areas, and 40.1% for control 2 areas). The SAS SURVEYSELECT procedure was used to obtain simple random samples of control subjects by sex, age, and education group. The numbers of controls were not exactly four times the number of cases because there were insufficient controls in matching pools (Table 1).

Distributions of matched variables and covariates were examined and then logistic regression was performed against exposure status with covariates similar to the ones used for the Cox models. Stratified analyses for two sex groups and four birth cohort groups were performed to examine the heterogeneity of effects.

For the sensitivity analyses, models were examined with six different sets of covariates, and also by comparing the results obtained from the survival analyses with those from logistic regressions.

### 2.3. Review of Detection Bias Potential

We attempted to identify any potential differences in cancer-screening activities by exposure status by examining the following three sources of information: (1) frequencies of radiation-related medical tests; (2) frequencies of medical utilization based on health insurance statistics; and (3) records of health check services from the annual reports of the Korea Hydro and Nuclear Power Plant Companies and the National Cancer Center Investigation Report.

## 3. Results

The overall cancer incidence rate, in the form of ASR, was similar to that estimated by the original study. Compared to the unexposed group (combined control 1 and control 2 groups), the ASRs of the exposed group were higher for stomach cancers and thyroid cancers in both males and females, for liver cancers in males only, and for female breast cancers. The ASRs for radiation-related cancers as well as total cancers of the exposed group were also higher than those of the unexposed group (Table 2). For the SIR, direct comparisons between three groups are inappropriate as the sex and age distributions are not exactly the same. However, the overall findings are more or less the same with the ASR (Table 2), and compared to the national reference, significantly elevated SIRs were noted for all cancers of males, stomach cancers of both males and females, male liver cancers, and thyroid cancers of both males and females of the exposed group (see Appendix A).

### 3.1. Thyroid Cancer

The risks of developing thyroid cancers in females increased significantly in the exposed and control 1 groups in the survival and logistic models. For males, the risk was elevated but marginally significant in the survival analysis, with wider confidence intervals (Table 3).

**Figure 1:**
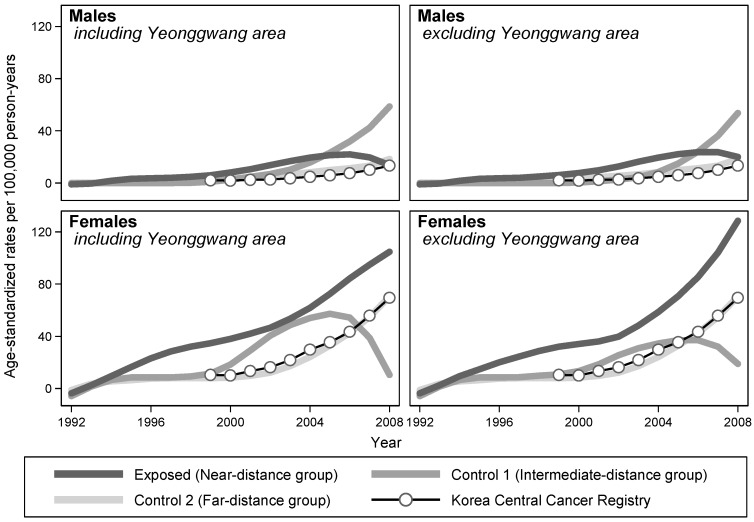
Trends of thyroid cancer incidences by distance of residence from NPPs in South Korea, 1992–2008. The estimates of the rates (exposed, control 1, and control 2) were smoothed by applying the locally weighted scatterplot smoothing (LOWESS) algorithm with a bandwidth of 0.8.

The annual ASRs of three groups were plotted over calendar years of follow-up using the LOWESS method. For females, the ASRs of the exposed group were higher than those of the unexposed group throughout the follow-up period, regardless of whether the records of Yeonggwang County were excluded. For males, the ASRs of the exposed group were also elevated throughout, except for the last 3 years, when the ASRs of the control 1 group caught up to the increasing trend (Figure 1).

The mean ages at the time of enrollment and the end of the observation were similar among the three groups. In addition, the mean ages of participants when they began to live around NPPs were similar between the exposed and control 1 groups. When we examined thyroid cancer patients only, the mean ages at the beginning of residence and at the time of diagnosis were lower for the exposed group than for the control 1 group.

As the recruitment of study participants began long after NPPs had begun operating, most of the participants living near NPPs had been exposed to them for considerable years at the time of enrollment. We divided the cohort members into three groups based on the potential length of exposure before enrollment (<10 years, 10–14 years, and >15 years), as also performed in the original study. The group with the longest period of exposure before enrollment was the oldest at the time of enrollment; the mean ages were 42.3, 50.4, and 59.2 years old for males, and 37.8, 50.9, and 60.0 years old for females, respectively. The group with longest exposure also had the shortest follow-up periods; the average follow-up durations were 13.7, 10.4, and 6.5 years for males, and 11.6, 8.8, and 6.6 years for females, respectively.

If screening out preexisting cancer patients had any effect on risk estimation for the cohorts, such an effect will be most likely for the oldest participants at the time of enrollment, and least likely to be corrected for the participants with the shortest observation. On the other hand, if screening out participants has no effect, then there should be no differences in ASRs between groups of different exposure periods before enrollment. Among the three groups with different exposure durations before enrollment, we observed the lowest ASRs for the longest period of exposure in both males and females, who were the oldest at the time of enrollment and had the shortest observation time.

When we divided the study participants into four birth cohort groups, the length of exposure was found to be protective or positive but not significant for the older birth cohorts. However, for the younger birth cohorts, living near NPPs was associated with an elevated risk of thyroid cancer, especially among females (Table 4).

### 3.2. Female Breast Cancer

**Figure 2:**
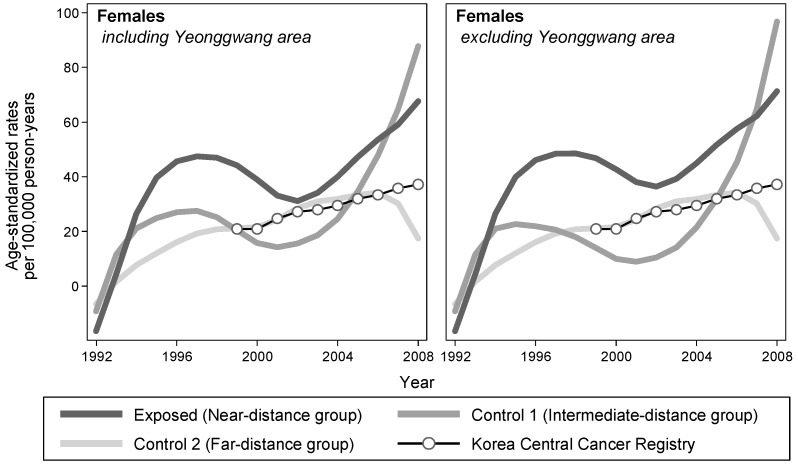
Trends of female breast cancer incidences by distance of residence from NPPs in South Korea, 1992–2008. The estimates of the rates (exposed, control 1, and control 2) were smoothed by applying the LOWESS algorithm with a bandwidth of 0.8.

The risk of breast cancer was elevated among the exposed group, but the difference was not statistically significant in both prospective cohort and case-control analyses (Table 3). Examining the temporal trend of ASRs by exposure status, the LOWESS lines of the exposed group were consistently elevated compared with those of the control groups, throughout the observation period (Figure 2).

Analyses by stratification of the four birth cohorts showed that the effect sizes became larger for the younger birth cohorts in both prospective and case-control analyses. The effect estimates for the youngest cohort became almost significant (Table 4).

### 3.3. Radiation-Related Cancers

**Figure 3:**
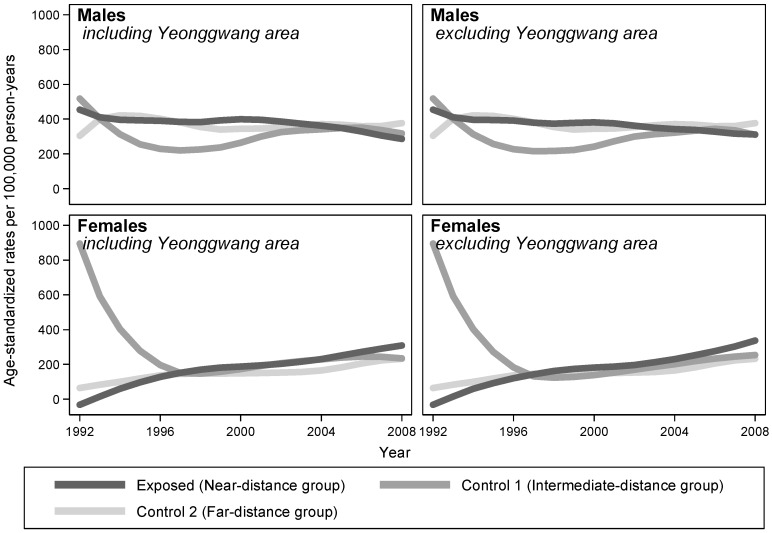
Trends of radiation-related cancer incidences by distance of residence from NPPs in South Korea, 1992–2008. The estimates of the rates (exposed, control 1, and control 2) were smoothed by applying the LOWESS algorithm with a bandwidth of 0.8.

The overall risks for radiation-related cancers were not significantly different between the exposed and unexposed groups (Table 3). Examining the temporal trends of ASRs, the LOWESS plots considerably overlapped between comparison groups in both males and females (Figure 3).

As for the analyses stratified by birth cohort, the effects of exposure on radiation-related cancers were somewhat protective for older cohorts, while the risk estimates became larger for the younger cohorts. However, when repeating the same birth cohort analysis for radiation-insensitive cancers, exposure had neither a protective effect in the older cohort groups nor a particularly harmful effect in the younger cohorts (Table 5).

### 3.4. Evaluation of Potential Detection Bias

Based on the original report [9], there were no differences in the frequencies of medical tests such as simple chest X-rays between the exposed and unexposed groups, while hospital visits were less frequent among the exposed group. In terms of medical service utilization, there were also no differences between the comparison groups (see Appendix A).

In Yeonggwang County, one of the four NPP sites, suspicious clusters of thyroid cancers were identified in 2006 by the National Cancer Center of Korea. Further investigation of these clusters revealed that intensive thyroid scans had been undertaken by two local practitioners during the early 2000s [10]. However, when we located the home addresses of the thyroid cancer patients who had received such screenings, most of them were more than 5 km from the NPP and belonged to the control 1 group rather than the exposed group. The same was true when we examined the locations of communities where voluntary health check services were provided by the Korea Hydro and Nuclear Power Plant Companies [11]. Based on their reports, screening activities seemed to be more common in the control 1 areas than in the exposed areas.

## 4. Discussion

In a prospective cohort study of cancer-screened adults at the time of enrollment, the original authors regarded the apparently elevated risk of female thyroid cancers near to nuclear power plants as irrelevant based on lower risk profile for longer pre-enrollment exposure duration and concluded that there was no need for further epidemiologic study. In this reanalysis, we replicated the original study’s results and conducted additional analyses to examine the underlying features of associations that could help interpret the results with more nuance. As epidemiologic findings are based on observation of (randomly) selected (or sampled) subjects, the process of selection and the nature of observation can influence results and their interpretation in many ways.

Four topics are further discussed in the following, including selection effects of pre-existing cancer patients at the time of enrollment, detection bias potential for residents living near NPPs by differential uses of thyroid scans, fluctuation of radiation waste discharge over time and its potential effects on outcomes, and consistencies or discrepancies with other countries’ experiences.

### 4.1. Exclusion of Pre-Existing Cancer Patients from the Cohort Enrollment

In an ideal cohort study, population at risk of target diseases should be enrolled and be followed up in order to identify the development of such diseases. However, what happens if study enrollment begins long after the suspected exposure began and enough time for disease development has already passed? In our analysis, if residing near NPPs does not increase cancer risk, then there should be no difference in risks between the exposed and unexposed in all age groups, regardless of age at the time of enrollment. When we divided the entire cohort into four different birth cohorts based on calendar year of birth, however, we observed significant differences in risks between the comparison groups, mainly in the younger cohort members, but not in the older. This was especially true for thyroid cancers, female breast cancers, and radiation-related cancers, but not for radiation-insensitive cancers.

The presence of differences in risk in younger but not older birth cohorts fits with the effect of screening-out pre-existing cancer cases in the cohort analysis results. Since participants who had been diagnosed with cancer at the time of entry into the cohort were removed from the analysis, the remaining subjects who were old enough to have passed the peak years of age for cancer development were actually survivors and less likely to be diagnosed with cancer even when continuously exposed and followed up. In contrast, the younger participants who were not old enough to be screened out at the time of enrollment would eventually show higher risks if the exposure really did increase risk.

Indeed, considering that children and adolescents are more sensitive to radiation [12], any study on middle-aged and elderly individuals free of cancer even long after potential exposure is likely to be biased toward the null. Therefore, the overall picture of cancer development for different areas and for different birth cohorts is consistent with specific radiation-related effects, modified by the screening-out of exposure-related cancers among those who had passed the age of peak cancer development at the time of cohort enrollment.

### 4.2. Detection Bias

We attempted to examine the possibility of detection bias that may be present for nearby residents, as NPPs had provided voluntary health check services to nearby communities. In addition, a huge increase in thyroid cancer incidence has been reported in Korea, which was ascribed mainly to indiscriminate ultrasound thyroid scanning by practitioners [13,14].

Screening for thyroid cancer by ultrasound scanners became widespread in Korea around 2000. However, when we examined the ASRs of thyroid cancer over years between the exposed and unexposed groups, the differences began to emerge soon after the start of follow-up in 1992 and persisted throughout 1990s, during which time the screening pressure should have been minimal with ultrasound thyroid scans not yet available. Additionally, when we analyzed data for three NPP sites, excluding Yeonggwang County where intensive screening had occurred, the differences in the ASRs of thyroid cancer between the exposed and control 1 groups increased instead.

In fact, when we compared the frequencies of medical tests such as simple chest X-rays between the exposed and unexposed groups, there were no differences, and hospital visits were less frequent among the exposed. In terms of medical service utilization, there were no differences. We concluded that detection bias towards the exposed group was unlikely. Considering the level of accessibility to medical services nationwide and the size and volume of medical examination schemes of national and local governments, the potential for detection bias confined to nearby residents seems highly unlikely [15].

### 4.3. Unscheduled Stoppages of Operations and Uncontrolled Discharges of Waste into the Environment in the Early Period of NPP Operation

The official reports of NPP waste discharge into the environment were not readily available. We had to request relevant information through a ‘Freedom of Information Act of Korea’ to get as-yet unpublished data. By reviewing a report written in the early periods of operation of the first NPP at Kori [16], we could identify unscheduled stoppages and uncontrolled discharges of waste into the environment.

In the nested case-control study, when thyroid cancer patients were compared with control individuals of the same age and sex within the exposed areas, the durations of residence of the cases were much longer than those of controls. In addition, whether or not they resided in the area during the early periods of the NPP was different between cases and controls. Likewise, the ages at enrollment and diagnosis were younger for cases living near NPPs than for those in control areas, even though the ages at enrollment and last observation were similar between the exposed and unexposed groups.

### 4.4. Comparison with Other Countries’ Experiences

The international nuclear event scale classifies events in the operation of NPPs into seven classes; those equal to or more severe than class 4 are classified as accidents and those less severe than class 4 are classified as incidents. Since 1990, in total 49 incidents were reported in Korea, five of which were shut-down incidents. When the frequency of these incidents was adjusted by the number and operation years of NPPs in Korea, the adjusted frequency of incidents was 0.127 incidents/year/plant (4–12 times higher than those for US, UK, Canada, France, and Germany) [17]. The same was true for shut-down incidents.

In the meta-analysis of thyroid cancers around NPPs, among good or adequate quality studies, a significant increase was reported only for NPPs located in Asia, that is, in Korea, as opposed to in European or North American sites [18]. Similarly, a significant increase in thyroid cancer was noted only if exposure was defined by specific distances such as 20 km instead of with vague terms like ‘living nearby’. In this meta-analysis, the existence of elevated thyroid cancer risks for people residing near NPPs only in Korea may not be an aberrant finding when the adjusted frequency of NPP incidents was accounted for, along with the well-established nationwide health care delivery system of Korea.

### 4.5. Limitations of the Reanalysis and Directions for Future Studies

This study was the first ever attempt at reanalysis of a major epidemiologic study in Korea. Before the reanalysis, several rounds of correspondence were exchanged about the shortcomings and interpretations of the results, including comparability of control group especially due to the differences in education status, potentials of selection bias from excluding cancer patients of already exposed, problems of mechanical selection processes for covariates in multivariate modelling, interpretation of the gender differences, and study design limits of targeting adults only population [2,3,4,5]. However, most of the problems stemming from the design could not be solved by the reanalysis, but only checked for their potentials. We could neither fully replicate the original study nor examine systematic differences between participants and non-participants because the entire set of variables and information of non-participants was not provided.

Nevertheless, the overall results of the reanalysis indicate that the conclusions of the original study were inappropriate. Not only females, but also males, had an elevated risk of thyroid cancers, and increased risks of breast cancers and radiation-related cancers were observed, especially among younger cohorts. These findings are quite compatible with the increased risk of radiation-related cancers among people residing near NPPs. The main reason for these quite different conclusions to the original article was the different strategies used to control the effects of screening-out prevalent cancer cases at enrollment; in this study, stratified analysis based on calendar year of birth was conducted to examine the effects of age at screening on the follow-up results.

This kind of difference clearly shows the importance of reanalysis of major epidemiologic studies. However, there have been controversies regarding reanalysis, including the issue of data ownership. The most difficult problem in the reanalysis was encountered when the arguments about falsely-claimed copyright and the right-to-know of the public domain dataset clashed with each other. In the future, reanalysis procedures should be specified in detail, especially for those studies that are supported by public funds over a long period of time.

## 5. Conclusions

A 20-year follow-up study on cancer incidence among people living near NPPs in South Korea ended in 2011 with a finding of significantly elevated thyroid cancer risk for females (HR 2.5). Original authors had disregarded the findings of male thyroid cancers based on statistical non-significance, and treated the elevated female thyroid cancer risk as irrelevant based on inconsistent findings of lower risks for longer residence groups.

Upon reanalysis of four subcohorts based on calendar year of birth, the statistically significant thyroid cancer risks of females living near nuclear power plants became higher in younger birth subcohorts (HR 3.38). Even though statistically of marginal significance, this was also true for male thyroid cancers (HR 1.74), female breast cancers (HR 2.24) and radiation-related cancers (HR 1.59 for males, HR 1.77 for females), but not for radiation-insensitive cancers (HR 0.59 for males, HR 0.98 for females). 

The follow-up of the original study started in 1992, long after the beginning of NPPs operation in 1978, and only those adults who remained healthy throughout residence were recruited for follow-up after excluding prevalent cancer cases at the enrollment. If residing near to NPPs has no effects on cancer incidence, then the recruiting survivors after screening will not affect the comparison between exposed and controls across for different age groups. However, if NPPs do have effects on cancer development, then the NPP effects will be screened less likely for the younger subcohorts who have not yet reached the peak age of cancer incidence, and the NPPs effects between exposed and controls will get stronger for the younger subcohorts during follow-up. 

The overall reanalysis results of the elevated risks for radiation-sensitive, but not for radiation-insensitive cancers, getting stronger among the younger subcohorts, are quite compatible with the radiation effects of NPPs. We found that the inconsistent relations between the length of residence and the thyroid cancer risks were due to the cancer screening of longer residence groups who were in fact older in age at enrollment and followed up for shorter period. Instead, we noticed that, the residence histories of thyroid cancers in exposed area were different from those of control area. 

The potential of detection bias or overdiagnosis in explaining the increase in cancer risk among exposed was unlikely because of the following two reasons; there were no differences in the frequencies of diagnostic tests or medical services between exposed and controls, and the increase in thyroid cancer among exposed was evident throughout the whole follow-up period from 1992 to 2008, while the overdiganosis of thyroid cancer began to emerge on the nationwide statistics only after 2000 in Korea with the spread of ultrasound scan. 

On the other hand, when we examined the radiation discharge from NPPs since the beginning of their operation, we have found evidences of environmental irregularities including the unscheduled releases of excessive levels of radioactive wastes in the early years (1978~1980) of NPP operation, and the much higher (4 to 12 times) reported frequency of nuclear event incidents for NPPs of Korea compared to US, UK, Canada, France or Germany. 

All these results of reanalysis of the original study indicate the consistent effects of environmental exposures from NPPs on the increased cancer risks among residents in Korea, while there were no consistent evidences of detection bias. In the future, all the studies that are supported by public funds over a long period of time should be open to reanalysis, and its procedures should be specified in detail from the beginning. 

## Figures and Tables

**Table 1:** Numbers of cases and controls for nested-case analysis.

Cancer Group	Nested Cases	Matched Controls
Thyroid	125	500
Female breast	78	313
Male stomach	229	916
Male liver	198	789
Radiation-related cancers ^a^	1613	6420
Radiation-insensitive cancers	684	2725
Total cancers	2297	9044

^a^ International Agency for Research on Cancer (IARC) Group 1 definition of radiation-related cancers: brain (central nervous system), bone, skin (excluding melanoma), nose and sinus, lung and airway, thyroid, breast, salivary gland, esophagus, stomach, colon and rectum, liver and biliary tract, kidney, bladder, leukemia (excluding chronic lymphocytic leukemia).

**Table 2:** Cancer incidence rates (/100,000) by exposure status and gender.

Cancer Sites	Exposure Status ^a^	Male			Female		
		No.	Crude Rates	ASRs ^b^	No.	Crude Rates	ASRs
All	Exposed	393	904.4	566.2	312	541.1	307.0
	Unexposed	941	1093.4	545.2	651	560.4	281.2
	Control 1	421	1048.3	509.9	300	618.7	306.5
	Control 2	520	1132.8	567.9	351	518.7	264.0
Radiation-related	Exposed	251	572.9	358.6	179	308.3	189.3
	Unexposed	580	667.7	343.1	366	313.2	161.2
	Control 1	249	614.4	295.8	172	352.2	181.5
	Control 2	331	714.3	375.0	194	285.3	146.5
Stomach	Exposed	101	229.2	140.7	61	104.4	49.5
	Unexposed	191	218.7	104.0	128	109.0	51.1
	Control 1	78	191.4	96.0	64	130.3	58.8
	Control 2	113	242.5	110.3	64	93.7	44.5
Liver	Exposed	67	151.4	97.6	13	22.2	9.5
	Unexposed	131	149.4	75.6	51	43.3	17.7
	Control 1	53	129.5	64.9	16	32.5	13.1
	Control 2	78	166.8	85.6	35	51.1	21.1
Thyroid	Exposed	10	22.6	15.9	44	75.3	60.7
	Unexposed	14	15.9	11.4	57	48.5	33.0
	Control 1	11	26.8	16.3	30	61.0	43.1
	Control 2	3	6.4	8.2	27	39.5	26.4
Breast	Exposed				33	56.5	44.7
	Unexposed				45	38.3	29.7
	Control 1				20	40.6	30.3
	Control 2				25	36.3	29.0

^a^ Exposed: residing <5 km from nuclear power plants (NPPs), Unexposed: combining control 1 and control 2, Control 1: residing 5–30 km from NPPs, Control 2: residing >30 km from NPPs; ^b^ ASR: Age-standardized rates.

**Table 3:** Adjusted hazard ratios and odds ratios of exposed group compared to control 2 group for thyroid, breast, and radiation-related cancers by gender.

Cancer	Exposure Status ^a^	Male		Female	
		Hazard Ratio (95% CI ^b^)	Odds Ratio (95% CI)	Hazard Ratio (95% CI)	Odds Ratio (95% CI)
Thyroid	Exposed	3.38 (0.92–12.39)	4.33 (1.10–17.24)	3.15 (1.56–6.34)	2.06 (1.21–3.51)
	Control 1	4.10 (1.14–14.73)	5.30 (1.35–20.79)	2.26 (1.08–4.72)	1.58 (0.89–2.80)
	Control 2	Ref.	Ref.	Ref.	Ref.
Breast	Exposed			1.47 (0.77–2.80)	1.62 (0.90–2.89)
	Control 1			1.26 (0.63–2.52)	1.16 (0.61–2.23)
	Control 2			Ref.	Ref.
Radiation-related	Exposed	1.01 (0.81–1.26)	0.90 (0.75–1.07)	1.10 (0.89–1.36)	1.18 (0.97–1.44)
	Control 1	0.94 (0.76–1.16)	1.09 (0.92–1.30)	1.17 (0.95–1.45)	1.29 (1.06–1.58)
	Control 2	Ref.	Ref.	Ref.	Ref.

^a^ Exposed: residing <5 km from NPPs, Unexposed: combining control 1 and control 2, Control 1: residing 5–30 km from NPPs, Control 2: residing >30 km from NPPs; ^b^ CI: Confidence interval.

**Table 4:** Adjusted hazard ratios and odds ratios for female thyroid and breast cancers by birth cohort.

Risk Estimates	Exposure Status ^a^	Birth Year			
		1901–1934	1935–1942	1943–1955	1956–1984
Thyroid cancer					
Hazard ratios (95% CI ^b^)	Exposed	0.48 (0.04–6.18)	7.01 (0.85–57.46)	3.60 (1.21–10.73)	3.38 (0.94–12.20)
	Control 1	0.49 (0.04–6.48)	9.51 (1.21–74.91)	1.14 (0.32–4.05)	3.72 (0.96–14.38)
	Control 2	Ref.	Ref.	Ref.	Ref.
Odds ratio (95% CI)	Exposed	0.27 (0.03–2.68)	2.16 (0.58–8.07)	2.93 (1.23–6.99)	2.13 (0.84–5.36)
	Control 1	0.90 (0.18–4.64)	4.23 (1.24–14.72)	1.12 (0.39–3.25)	1.23 (0.43–3.47)
	Control 2	Ref.	Ref.	Ref.	Ref.
Breast cancer					
Hazard ratio (95% CI)	Exposed	0.68 (0.13–3.44)	1.75 (0.44–7.07)	1.25 (0.35–4.50)	2.24 (0.72–6.91)
	Control 1	0.89 (0.21–3.84)	0.70 (0.14–3.61)	1.23 (0.33–4.60)	1.92 (0.54–6.88)
	Control 2	Ref.	Ref.	Ref.	Ref.
Odds ratio (95% CI)	Exposed	1.69 (0.33–8.73)	1.17 (0.36–3.84)	1.10 (0.36–3.42)	2.61 (0.96–7.06)
	Control 1	2.44 (0.49–12.01)	0.64 (0.15–2.82)	1.11 (0.34–3.62)	1.21 (0.37–3.93)
	Control 2	Ref.	Ref.	Ref.	Ref.

^a^ Exposed: residing <5 km from NPPs, Unexposed: combining control 1 and control 2, Control 1: residing 5–30 km from NPPs, Control 2: residing >30 km from NPPs; ^b^ CI: Confidence interval.

**Table 5:** Adjusted hazard ratios and odds ratios for radiation-related and radiation-insensitive cancers by gender and birth cohort.

Risk Estimates	Exposure Status ^a^	Male				Female			
		Birth Year				Birth Year			
		1901–1934	1935–1942	1943–1955	1956–1984	1901–1934	1935–1942	1943–1955	1956–1984
Radiation-related cancers									
Hazard ratio (95% CI ^b^)	Exposed	1.04 (0.75–1.44)	0.88 (0.60–1.30)	1.20 (0.66–2.18)	1.59 (0.49–5.15)	0.78 (0.55–1.10)	1.14 (0.77–1.70)	1.43 (0.88–2.32)	1.77 (0.94–3.32)
	Control 1	0.99 (0.73–1.35)	0.86 (0.60–1.24)	0.91 (0.49–1.68)	1.05 (0.30–3.68)	1.02 (0.74–1.41)	1.18 (0.80–1.74)	1.18 (0.71–1.97)	2.06 (1.04–4.08)
	Control 2	Ref.	Ref.	Ref.	Ref.	Ref.	Ref.	Ref.	Ref.
Odds ratio (95% CI)	Exposed	0.80 (0.61–1.04)	0.80 (0.59–1.08)	1.28 (0.81–2.01)	1.68 (0.79–3.58)	0.96 (0.69–1.32)	1.08 (0.74–1.58)	1.33 (0.84–2.09)	2.19 (1.25–3.85)
	Control 1	1.23 (0.96–1.57)	0.92 (0.68–1.24)	1.14 (0.71–1.83)	0.92 (0.38–2.22)	1.33 (0.98–1.81)	1.35 (0.93–1.95)	1.18 (0.73–1.91)	1.18 (0.62–2.26)
	Control 2	Ref.	Ref.	Ref.	Ref.	Ref.	Ref.	Ref.	Ref.
Radiation-insensitive cancers									
Hazard ratio (95% CI)	Exposed	1.33 (0.86–2.05)	1.30 (0.80–2.10)	1.19 (0.66–2.16)	0.59 (0.20–1.73)	0.70 (0.42–1.17)	0.79 (0.38–1.64)	1.54 (0.53–4.41)	0.98 (0.32–2.97)
	Control 1	1.21 (0.80–1.83)	1.19 (0.75–1.91)	0.78 (0.41–1.47)	0.34 (0.08–1.37)	0.53 (0.30–0.91)	0.82 (0.40–1.66)	1.64 (0.58–4.64)	1.05 (0.31–3.54)
	Control 2	Ref.	Ref.	Ref.	Ref.	Ref.	Ref.	Ref.	Ref.
Odds ratio (95% CI)	Exposed	1.23 (0.81–1.87)	1.02 (0.65–1.61)	1.40 (0.81–2.41)	1.35 (0.51–3.59)	0.95 (0.60–1.51)	0.92 (0.47–1.81)	1.26 (0.59–2.71)	1.27 (0.47–3.42)
	Control 1	1.61 (1.08–2.40)	1.19 (0.76–1.87)	1.03 (0.57–1.87)	0.41 (0.10–1.62)	0.76 (0.46–1.26)	1.09 (0.56–2.12)	1.68 (0.80–3.54)	0.81 (0.27–2.48)
	Control 2	Ref.	Ref.	Ref.	Ref.	Ref.	Ref.	Ref.	Ref.

^a^ Exposed: residing <5 km from NPPs, Unexposed: combining control 1 and control 2, Control 1: residing 5–30 km from NPPs, Control 2: residing >30 km from NPPs; ^b^ CI: Confidence interval.

## Appendix A

**(1) Locations of the study areas**

**(2) Distributions and Treatments of those lost to follow-up**

In the original study, follow-up was carried out only once at the end of the study, and no information about when and where about was available for those lost-to-follow-ups (LTFUs). In our analysis, LTFUs without any evidence of follow-up were removed from the dataset, as we could not determine the time of LTFU, for survival analysis.

When we examined the distribution of LTFU across three study groups, there was no significant differences in LTFU by exposure status (Table A1).

**Table A1:** Distribution of participants lost to follow-up by exposure status (*n* = 36,176).

Sub-Cohort Groups	Male, *n* (%)			Female, n (%)		
	LTFU (*n* = 60)	Not LTFU (*n* = 14,934)	*p* Value	LTFU (*n* = 90)	Not LTFU (*n* = 21,092)	*p* Value
Exposed	21 (0.47)	4470 (99.53)	0.511	37 (0.54)	6839 (99.46)	0.192
Control-1	19 (0.43)	4424 (99.57)		20 (0.34)	5860 (99.66)	
Control-2	20 (0.33)	6040 (99.67)		33 (0.39)	8393 (99.61)	

LTFU: lost to follow-up. ‘Lost to follow-up’ is defined as “Subjects in a longitudinal study whose status or outcome is unknown.” by the definition of ‘A Dictionary of Epidemiology (sixth edition)’. *p* values were calculated with the use of the chi-square test.

**(3) SIRs of three exposure groups**

Out of the follow-up period of 1992 to 2008, nation-wide cancer incidence is available from 1999, and the standardized incidence ratio (SIR) based on indirect standardization with national reference was calculated only for the period of 1999 to 2008. For the SIR, direct comparisons between three groups are inappropriate as the sex and age distributions are not exactly the same. However, the overall findings are more or less the same as the ASR, and compared to the national reference, significantly elevated SIRs were noted for all cancers of males, stomach cancers of both males and females, male liver cancers, and thyroid cancers of both males and females of the exposed group (Table A2).

**Table A2:** Standardized cancer incidence ratios during the follow-up period of 1999–2008 by the exposure status and gender.

Cancer Sites	Exposure Status ^a^	Total		Male		Female	
		O/E ^b^	SIR ^c^ (95% CI ^d^)	O/E	SIR (95% CI)	O/E	SIR (95% CI)
All	Exposed	625/573.62	1.09 (1.01–1.18)	344/297.93	1.15 (1.04–1.28)	281/275.69	1.02 (0.91–1.15)
	Unexposed						
	Control 1	683/633.80	1.08 (1.00–1.16)	403/358.08	1.13 (1.02–1.24)	280/275.72	1.02 (0.90–1.14)
	Control 2	798/797.21	1.00 (0.93–1.07)	477/442.85	1.08 (0.98–1.18)	321/354.36	0.91 (0.81–1.01)
Radiation-related	Exposed	433/464.99	0.93 (0.85–1.02)	221/247.19	0.89 (0.78–1.02)	212/217.80	0.97 (0.85–1.11)
	Unexposed	1048/1156.94	0.91 (0.85–0.96)	609/659.73	0.92 (0.85–1.00)	439/497.21	0.88 (0.80–0.97)
	Control 1	486/513.18	0.95 (0.87–1.04)	272/295.48	0.92 (0.82–1.04)	214/217.70	0.98 (0.86–1.12)
	Control 2	562/643.76	0.87 (0.80–0.95)	337/364.25	0.93 (0.83–1.03)	225/279.50	0.81 (0.71–0.92)
Stomach	Exposed	146/110.99	1.32 (1.12–1.55)	88/67.25	1.31 (1.06–1.61)	58/43.74	1.33 (1.03–1.72)
	Unexposed	286/279.94	1.02 (0.91–1.15)	174/178.92	0.97 (0.84–1.13)	112/101.02	1.11 (0.92–1.33)
	Control 1	130/125.07	1.04 (0.88–1.23)	73/80.10	0.91 (0.72–1.15)	57/44.97	1.27 (0.98–1.64)
	Control 2	156/154.87	1.01 (0.86–1.18)	101/98.82	1.02 (0.84–1.24)	55/56.05	0.98 (0.75–1.28)
Liver	Exposed	73/61.79	1.18 (0.94–1.49)	61/41.39	1.47 (1.15–1.89)	12/20.40	0.59 (0.33–1.04)
	Unexposed	169/152.89	1.11 (0.95–1.29)	123/104.88	1.17 (0.98–1.40)	46/48.01	0.96 (0.72–1.28)
	Control 1	64/68.74	0.93 (0.73–1.19)	50/47.34	1.06 (0.80–1.39)	14/21.39	0.65 (0.39–1.10)
	Control 2	105/84.15	1.25 (1.03–1.51)	73/57.54	1.27 (1.01–1.60)	32/26.61	1.20 (0.85–1.70)
Thyroid	Exposed	51/33.57	1.52 (1.15–2.00)	9/4.20	2.14 (1.12–4.12)	42/29.37	1.43 (1.06–1.93)
	Unexposed	69/73.78	0.94 (0.74–1.18)	14/9.54	1.47 (0.87–2.48)	55/64.24	0.86 (0.66–1.12)
	Control 1	40/30.87	1.30 (0.95–1.77)	11/4.32	2.54 (1.41–4.59)	29/26.54	1.09 (0.76–1.57)
	Control 2	29/42.92	0.68 (0.47–0.97)	3/5.22	0.57 (0.19–1.78)	26/37.70	0.69 (0.47–1.01)
Breast	Exposed	26/30.56	0.85 (0.58–1.25)	0/0.22	-	26/30.35	0.86 (0.58–1.26)
	Unexposed	44/66.28	0.66 (0.49–0.89)	2/0.57	3.49 (0.87–13.97)	42/65.70	0.64 (0.47–0.87)
	Control 1	19/27.42	0.69 (0.44–1.09)	1/0.26	3.87 (0.55–27.47)	18/27.16	0.66 (0.42–1.05)
	Control 2	25/38.86	0.64 (0.43–0.95)	1/0.31	3.19 (0.45–22.61)	24/38.54	0.62 (0.42–0.93)

^a^ Exposed: residing <5 km from NPPs, Unexposed: combining control 1 and control 2. Control 1: residing 5–30 km from NPPs, Control 2: residing >30 km from NP; ^b^ O: observed case number, E: expected case number; ^c^ SIR: standardized incidence ratio; ^d^ CI: confidence interval. We have conducted indirect standardization of the incidence rates based on the rates for South Korea in 1999–2008.

**(4) Comparison of medical service utilization across different exposure status**

Medical services including radiation therapy, nuclear medicine tests, and CT/fluoroscopic examinations as well as X-ray were tabulated by exposure status. The results showed that there were no differences in the utilization of those medical services (Table A3).

**Table A3:** The utilization frequency of medical tests by sex and exposure status.

Medical Tests	Frequency	Male (*n* (%))			Female (*n* (%))		
		Exposed ^a^	Control 1 ^b^	Control 2 ^c^	Exposed	Control 1	Control 2
Radiation Therapy	>10	5 (0.1)	3 (0.1)	5 (0.1)	2 (0.0)	6 (0.1)	8 (0.1)
	2~10	14 (0.3)	10 (0.2)	50 (0.8)	23 (0.3)	17 (0.3)	53 (0.6)
	1	12 (0.3)	6 (0.1)	36 (0.6)	25 (0.4)	19 (0.3)	67 (0.8)
	Never	4245 (95.0)	4127 (93.3)	5448 (90.2)	6479 (94.7)	5409 (92.3)	7491 (88.9)
	No Response	194 (4.3)	278 (6.3)	501 (8.3)	310 (4.5)	409 (7.0)	804 (9.6)
Nuclear Medicine Tests	>10	20 (0.5)	31 (0.7)	12 (0.2)	8 (0.1)	12 (0.2)	10 (0.1)
	2~10	154 (3.5)	166 (3.8)	156 (2.6)	184 (2.7)	160 (2.7)	166 (2.0)
	1	169 (3.8)	130 (2.9)	179 (3.0)	292 (4.3)	226 (3.9)	263 (3.1)
	Never	3910 (87.5)	3797 (85.8)	5183 (85.8)	6010 (87.9)	5028 (85.8)	7124 (84.9)
	No Response	217 (4.9)	300 (6.8)	510 (8.4)	345 (5.0)	434 (7.4)	830 (9.9)
X-ray, CT, Fluoro Tests	>10	940 (21.0)	974 (22.0)	1039 (17.2)	545 (8.0)	574 (9.8)	650 (7.7)
	2~10	2068 (46.3)	2112 (47.7)	2736 (45.3)	3999 (58.5)	3618 (61.7)	3850 (45.9)
	1	303 (6.8)	312 (7.1)	514 (8.5)	957 (14.0)	664 (11.3)	959 (11.4)
	Never	905 (20.3)	746 (16.9)	593 (9.8)	936 (13.7)	558 (9.5)	1015 (12.1)
	No Response	254 (5.7)	254 (6.3)	1158 (19.2)	402 (5.9)	446 (7.6)	1919 (22.9)

^a^ Exposed: residing <5 km from NPPs; ^b^ Control 1: residing 5–30 km from NPPs; ^c^ Control 2: residing >30 km from NP.

**(5) The results of male stomach cancer and male liver cancer analysis**

**Table A4:** Adjusted hazard ratios and odds ratios of exposed group compared to control 2 group for male stomach and liver cancers.

Cancer	Exposure Status ^a^	Male	
		Hazard Ratio (95% CI ^b^)	Odds Ratio (95% CI)
Male Stomach	Exposed	1.31 (0.92–1.86)	1.23 (0.93–1.63)
	Control 1	0.98 (0.68–1.40)	0.94 (0.68–1.31)
	Control 2	Ref.	Ref.
Male Liver	Exposed	0.99 (0.63–1.54)	1.19 (0.82–1.72)
	Control 1	0.74 (0.48–1.16)	0.92 (0.63–1.36)
	Control 2	Ref.	Ref.

^a^ Exposed: residing <5 km from NPPs, Unexposed: combining control 1 and control 2, Control 1: residing 5–30 km from NPPs, Control 2: residing >30 km from NPPs; ^b^ Confidence interval.
